# Cadherin Protein Is Involved in the Action of *Bacillus thuringiensis* Cry1Ac Toxin in *Ostrinia furnacalis*

**DOI:** 10.3390/toxins13090658

**Published:** 2021-09-15

**Authors:** Wenzhong Jin, Yuqian Zhai, Yihua Yang, Yidong Wu, Xingliang Wang

**Affiliations:** Key Laboratory of Integrated Pest Management on Crops in East China (MARA), College of Plant Protection, Nanjing Agricultural University, Nanjing 210095, China; 2018102105@njau.edu.cn (W.J.); 2020102093@stu.njau.edu.cn (Y.Z.); yhyang@njau.edu.cn (Y.Y.); wyd@njau.edu.cn (Y.W.)

**Keywords:** *Bacillus thuringiensis*, Asian corn borer, cadherin, CRISPR/Cas9, Cry1Ac resistance

## Abstract

Transgenic crops expressing *Bacillus thuringiensis* (Bt) insecticidal proteins have been extensively planted for insect pest control, but the evolution of Bt resistance in target pests threatens the sustainability of this approach. Mutations of cadherin in the midgut brush border membrane was associated with Cry1Ac resistance in several lepidoptera species, including the Asian corn borer, *Ostrinia furnacalis*, a major pest of maize in Asian–Western Pacific countries. However, the causality of *O. furnacalis* cadherin (*OfCad*) with Cry1Ac resistance remains to be clarified. In this study, in vitro and in vivo approaches were employed to examine the involvement of *OfCad* in mediating Cry1Ac toxicity. Sf9 cells transfected with *OfCad* showed significant immunofluorescent binding with Cry1Ac toxin and exhibited a concentration-dependent mortality effect when exposed to Cry1Ac. The *OfCad* knockout strain *OfCad*-KO, bearing homozygous 15.4 kb deletion of the *OfCad* gene generated by CRISPR/Cas9 mutagenesis, exhibited moderate-level resistance to Cry1Ac (14-fold) and low-level resistance to Cry1Aa (4.6-fold), but no significant changes in susceptibility to Cry1Ab and Cry1Fa, compared with the original NJ-S strain. The Cry1Ac resistance phenotype was inherited as autosomal, recessive mode, and significantly linked with the *OfCad* knockout in the *OfCad*-KO strain. These results demonstrate that the *OfCad* protein is a functional receptor for Cry1Ac, and disruption of *OfCad* confers a moderate Cry1Ac resistance in *O. furnacalis*. This study provides new insights into the mode of action of the Cry1Ac toxin and useful information for designing resistance monitoring and management strategies for *O. furnacalis*.

## 1. Introduction

The Gram-positive bacterium *Bacillus thuringiensis* (Bt) produces insecticidal toxins and has served as an excellent biological pesticide since its entomopathogenicity was discovered in the early 1900s [1]. To date, sprayable Bt formulations or transgenic Bt crops are the most effective alternatives to synthetic insecticides [2]. Major advantages of Bt crops driving their adoption include its high efficacy at suppressing devastating pests, increased crop yield and farmer gains, and reduced synthetic pesticide usage, thereby benefiting human and environmental health [3,4,5,6]. Unfortunately, evolution of resistance to Bt crops by target pests has reduced such benefits, and practical resistance has been documented in at least nine species [7,8,9].

The Asian corn borer (*Ostrinia furnacalis*), which is widely present in East and Southeast Asia, Australia, and the Western Pacific Islands, is a destructive pest of maize (*Zea mays*) [10]. The *O. furnacalis* larvae feed on nearly all tissues of maize and have caused severe yield losses ranging from 10% to 100% to maize in some countries of the Asian–Western Pacific region [10]. Feeding damage by the insect pest increases fungal infestation, which further influences the quality and commodity value of maize [11]. Although the insecticide resistance status of *O. furnacalis* is not serious in the field [12], current control of this pest primarily relies on insecticides. Furthermore, following the rapid spread of the fall armyworm (*Spodoptera frugiperda*) to the continents of Asia, the Pacific and Australia [13], extensive application of insecticides for emergency control might increase selection pressure on *O. furnacalis*. Therefore, undesirable consequences of pesticide use such as the evolution of resistance in insect pests, environmental pollution, and threats to human health should be carefully considered. An environmentally friendly and economically effective option for the control of major maize pests (*O. furnacalis* and *S. frugiperda*) is growing transgenic Bt maize. Bt maize was first commercially planted in 1996 and nowadays it is grown in 14 countries, and the global area has increased to 60.9 million hectares, including insect-resistant (Bt) and/or herbicide-tolerant transgenic maize [14].

In-depth study on the mode of action of Bt toxins is important for understanding how Bt exerts toxicity to insects, and for monitoring and managing the evolution of Bt resistance in target insects [15]. Current evidence indicates that activated Bt toxins bind via specific interactions with proteins expressed by midgut epithelial cells [16]. The mode of action of Bt is best studied for Cry1A toxins, which are primary Bt proteins used for lepidopteran pest control. A variety of proteins have been identified as putative or empirical functional receptors for Cry toxins, including alkaline phosphatase, aminopeptidase-N, cadherin and ATP-binding cassette (ABC) transporter family proteins [17]. Studies revealed that ABC transporters are critical for the Cry1A toxins to exert high toxicity, while the other receptors may further increase toxicity, thus playing an enhancing role [18,19]. Resistance to Cry1A in several insects is associated with alterations in the coding sequence or transcription of the genes encoding these midgut receptors [20,21,22,23,24,25].

Cadherin, a calcium ion binding protein, plays an essential role in cell adhesion, migration, cytoskeletal organization and morphogenesis [26]. The Cry1A-binding cadherins in insect midguts contain an extracellular domain consisting of 9 to 12 cadherin repeats (CRs), a membrane proximal region (MPR), a single transmembrane domain (TM) and a cytoplasmic domain (CYT). The roles of these domains in mediating the toxicity of Cry toxins vary: the extracellular domain, including the last CR and the MPR region, is involved in toxin binding and oligomerization, and the CYT domain may be responsible for signal transduction [27,28]. Diverse cadherin mutations associated with Cry1Ac resistance resulting in amino acid substitution, deletion or truncation were reported in *Heliothis virescens* [21], *Pectinophora gossypiella* [29,30,31,32,33,34] and *Helicoverpa armigera* [35,36,37,38,39,40]. However, the contribution of cadherin to Cry1Ac resistance may be insect species dependent. Genetic linkage analysis or functional studies have ruled out the involvement of cadherin in Cry1A resistance mechanisms in *Plutella xylostella* [41], *Trichoplusia ni* [42] and *S. frugiperda* [43].

In toxin selection studies, *O. furnacalis* evolved high-level resistance to single-use (Cry1Ac, Cry1Ab, Cry1Ah, Cry1Ie and Cry1F) or paired-use (Cry1Ab + Cry1F and Cry1Ab + Cry1Ie) toxins [44,45,46,47,48]. The evidence from laboratory conditions not only demonstrates that *O. furnacalis* can adapt to Cry selection pressure but also suggests the “pyramid strategy” did not consistently delay the development of resistance relative to the use of single Bt toxins. Therefore, a better understanding of the mechanisms underlying the *O. furnacalis* resistance to Cry toxins would provide new insights for developing Bt maize. Although Cry1Ac resistance in *O. furnacalis* was associated with the mutant and down-regulated cadherin [49], the causality of the *O. furnacalis* cadherin (*OfCad*) gene with Cry1Ac resistance, and the exact contribution of *OfCad* to resistance phenotypes, remains unknown.

In our previous study, gene knockout investigations demonstrated that a truncation of the *O. furnacalis* ABCC2 gene (*OfABCC2*) conferred a low level (*ca.* 8-fold) of resistance to Cry1Ac [50], which implied that OfABCC2 is involved, yet plays a minor role, in Cry1Ac mode of action. In this study, we employ cytotoxicity assays of Cry1Ac in Sf9 cell lines expressing *OfCad* and CRISPR/Cas9 genome editing approach to investigate the role that *OfCad* plays in the toxicity of Cry1Ac. Our findings indicate that cadherin of *O. furnacalis* is a functional receptor for the Cry1Ac, and knockout of *OfCad* confers moderate-level resistance to Cry1Ac in *O. furnacalis.*

## 2. Results

### 2.1. Recombinant OfCad Expression and Immunolocalization

Recombinant *OfCad* proteins were detected by Western blot analysis. A primary band for the protein samples was of the expected molecular mass (210 kDa, Figure S1). Corresponding bands were not detected in non-transfected Sf9 cells or empty bacmid (EB) transfected samples. The results indicated that the exogenous *O. furnacalis* cadherin proteins were successfully expressed in Sf9 cell lines.

**Figure 1:**
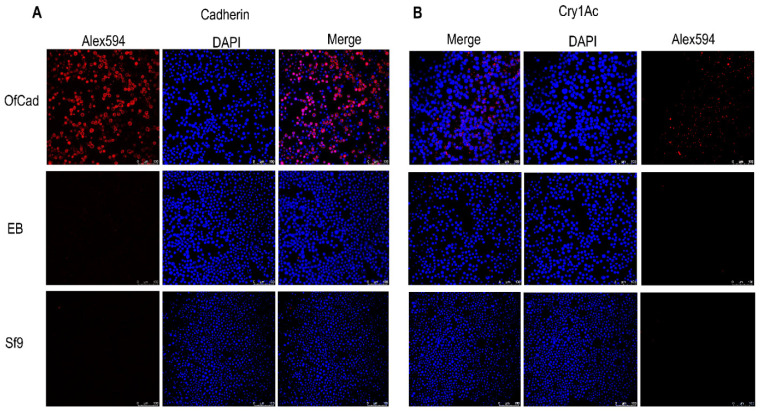
Immunolocalization of cadherin and Cry1Ac in non-transfected Sf9 cells (Sf9), Sf9 cells transfected by empty bacmid (EB) or by recombinant *OfCad*. (**A**) Immunofluorescence image of *OfCad*. Fixed Sf9 cells expressing *OfCad* proteins were probed with anti-His TagMouse monoclonal antibody, and fluorescence was detected by goat anti-mouse antiserum labeled with Alexa Fluor 594 (red). Cell nuclei were stained with DAPI (blue). (**B**) Immunofluorescence localization of Cry1Ac. The fixed Sf9 cells expressing *OfCad* proteins were incubated with Cry1Ac (200 nM) and then incubated with rabbit polyclonal anti-Cry1Ac antiserum. Immunofluorescence was detected by goat anti-rabbit antiserum labeled with Alexa Fluor 594 (red). Cells were examined using a Zeiss Laser scanning confocal microscope with 20 objective lens. Scale bars: 100 μm.

To locate the *OfCad* protein in Sf9 cells, immunolocalization assays were performed. Fluorescence patterns indicated that the protein was targeted to the cell membrane and was observed in *OfCad* transfected cells, while no or minimal fluorescence background was detected for non- or EB-transfected Sf9 cells (Figure 1A). Immunolocalization analyses between heterologously expressed *OfCad* protein and Cry1Ac were also performed and revealed significant Cry1Ac signal associated with cells expressing *OfCad*. In contrast, no fluorescence was detected in two negative control samples (non-transfected Sf9 cells and EB) (Figure 1B).

### 2.2. Toxicity of Cry1Ac to Sf9 Cells

**Figure 2:**
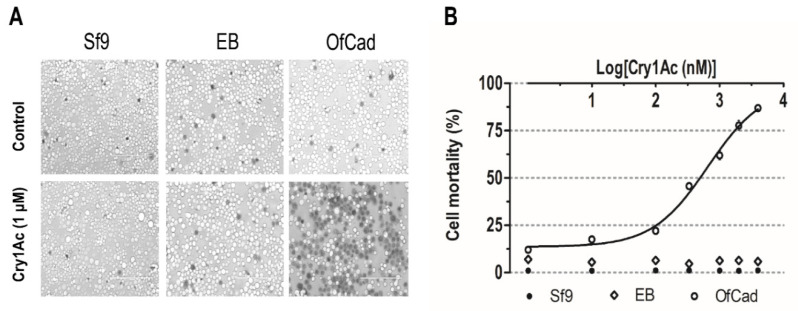
Cytotoxicity of Cry1Ac on non-transfected Sf9 cells (Sf9), Sf9 cells transfected by empty bacmid (EB) or by recombinant *OfCad*. (**A**) Imaging of Sf9 cells incubated with activated Cry1Ac (1 μM) or without toxin (Control). The cell lines were treated at 27 °C for 1 h, then stained with 0.4% trypan blue. The nuclei of cells that were live and therefore unstained at time of fixation are light colored, whereas dead or damaged cells dyed blue by the trypan vital stain are colored. A BX60 Olympus light microscope was used to examine cells. Scale bars: 20 μm. (**B**) Mortality of Sf9 cells exposed to several concentrations of Cry1Ac. Mortality is shown as the mean ± se of three repeated experiments, and a concentration–effect curve was visualized by GraphPad Prism 5.0 software (GraphPad Software Inc., San Diego, CA, USA).

After treatment with activated Cry1Ac ranging in concentration from 1 nM to 4 μM, Sf9 cells expressing recombinant *OfCad* became swollen and lysed. Mortality of Sf9 cells expressing recombinant *OfCad* increased with the increasing concentration of Cry1Ac (Figure 2), with the LC_50_ (concentration of causing half of the cells to swell) being 654.3 nM (95% FL: 512.3 to 835.7). In contrast, no significant swelling (mortality ≤1.2%) or low mortality (≤7.0%) was observed in non-transfected and EB-transfected cells, respectively (Figure 2B and Table S1). Thus, *OfCad* was found to mediate Cry1Ac against Sf9 cells, and the results indicated that *OfCad* is a functional receptor of Cry1Ac.

### 2.3. CRISPR/Cas9—Mediated OfCad Knockout in O. furnacalis

**Figure 3:**
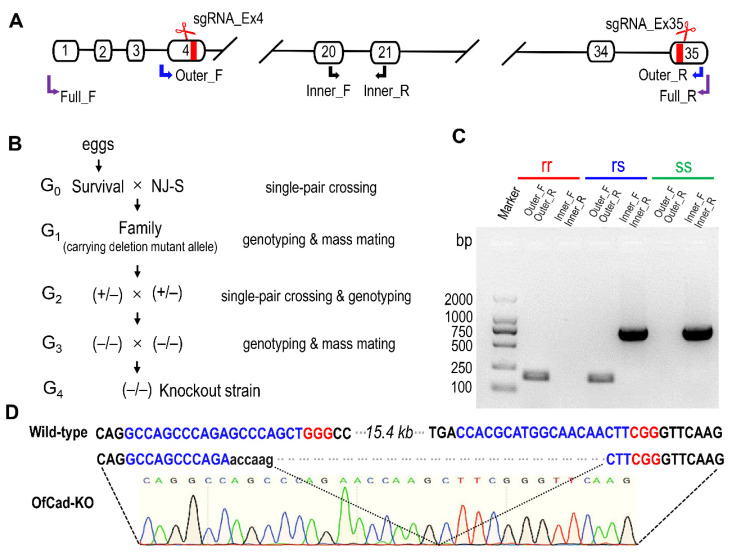
CRISPR/Cas9 mediated target mutagenesis of *OfCad* gene. (**A**) Diagram of *OfCad* gene structure, sgRNA targeting site and position of three pairs of primers for allele-specific PCR application. White boxes represent exons, and two sgRNA targeting sites were located at exon 4 and exon 35, respectively. (**B**) Crossing design for establishing homozygous *OfCad* gene knockout strain *OfCad*-KO. (**C**) Genotyping of individual *O. furnacalis* sample based on electrophoresis image pattern of allele-specific PCR products. ss, rs and rr represent the banding pattern of wild-type, heterozygous mutant and homozygous mutant, respectively. (**D**) Partial sequences of wild-type and manipulated mutant *OfCad* gene. The proto spacer sequence and protospacer adjacent motif (PAM) are shown in blue and red, respectively. Dual sgRNAs introduced 15.4 kb deletion *OfCad* in the *OfCad*-KO strain, which was confirmed by directly sequencing PCR products flanking the sgRNAs target regions, as shown in the chromatogram.

To construct an *O. furnacalis* cadherin knockout strain, 500 eggs of the susceptible NJ-S strain were injected with sgRNA_Ex4, sgRNA_Ex35 (Figure 3A) and Cas9 protein. Then, 100 injected eggs hatched, and 37 of them were reared to moths (G_0_). We made single-pair crosses between all the survivals (19 females, 18 males) and the NJ-S moths of opposite sex to produce 37 G_1_ families (Figure 3B). Next, 18 of the 37 families, which oviposited a high number of eggs, were selected for subsequent experiments. Twenty randomly collected, newly hatched larvae from each of the 18 G_1_ families were pooled as a mixed sample for DNA extraction. Based on the electrophoretogram of PCR products amplified by Outer_F and Outer_R primers (Figure 3A and Table S2), 5 G_1_ families were identified carrying CRISPR-mediated deletion alleles. One of the 5 G_1_ families that produced enough adults was used to mass cross to generate G_2_. A total of 384 larvae of G_2_ were treated with 1 μg/g Cry1Ac, and 40 survivals were obtained. Eight effective single-pair crosses among G_2_ were set up. After G_2_ oviposited, the genotypes of 16 parents were identified by banding patterns of two PCR products amplified by Outer_F/Outer_R and Inner_F/Inner R primers, respectively. None of the G_2_ family had both parents homozygous for the *OfCad* deletion, while three G_2_ families had both parents heterozygous for the *OfCad* deletion (+/− Figure 3B). We pooled the progeny from the three G_2_ families to produce G_3_. Subsequently, we excised one hind leg from a moth for NDA extraction and genotyping. Fifteen moths (9 females and 6 males) of 138 tested adults of G_3_ were homozygous for the *OfCad* deletion mutation (−/−, Figure 3B) and were pooled to mass mate in order to produce the *OfCad* knockout strain (G_4_), named *OfCad*-KO. Finally, 10 randomly selected individuals from the G_4_ (*OfCad*-KO strain) were genotyped as mutant homozygotes (Figure 3C, rr) and confirmed by direct sequencing the PCR products (Figure 3D).

### 2.4. Susceptibility of the OfCad-KO Strain to the Bt Toxins

When compared with the NJ-S strain, the susceptibility of *OfCad*-KO showed 14- and 4.6-fold reductions to Cry1Ac and Cry1Aa, respectively (Table 1). In contrast, Cry1Ab as well as Cry1Fa showed no significant differences in toxicity towards these two strains, with overlapped 95% fiducial limits of LC_50_s. The results indicate that the *OfCad* knockout confers moderate-level of resistance to Cry1Ac.

### 2.5. Dominance of Resistance to Cry1Ac in the OfCad-KO Strain

When treated with 1.0 µg/g of Cry1Ac, survival was 0% for NJ-S and 57.3% for *OfCad*-KO, respectively (Table 2). The F_1_ and F_1_′ progeny both had a 100% mortality under this concentration, and the dominance parameters (*h*) were both 0. These data show that the moderate Cry1Ac resistance phenotype was inherited in an autosomal, completely recessive mode in the *OfCad*-KO strain.

### 2.6. Genetic Association between the OfCad Knockout and Cry1Ac Resistance

**Figure 4:**
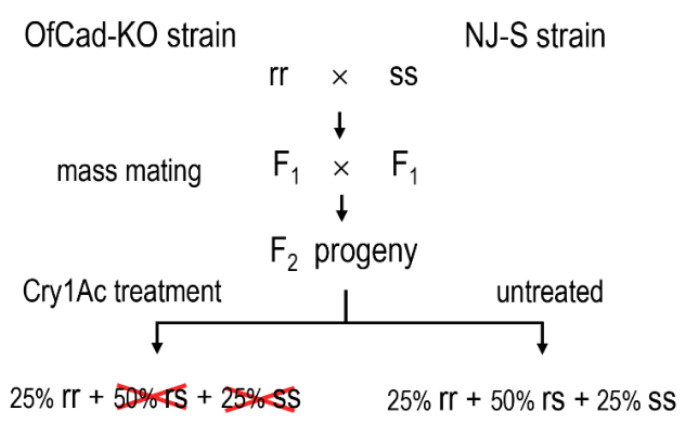
The schematic diagram depicts the mating process for linkage analysis between *OfCad* knockout and Cry1Ac resistance in the *O. furnacalis* *OfCad*-KO strain. rr, ss and rs represent samples bearing homozygous mutant, wild-type and heterozygous mutant *OfCad* allele, respectively.

Genetic crossing strategy (Figure 4) was employed to confirm the causality of the *OfCad* knockout with Cry1Ac resistance. Based on the electrophoresis image patterns of the target PCR products (Figure 3C), 30 individuals from NJ-S were homozygote carrying wild-type gene (ss), and 30 larvae from the *OfCad*-KO strain were homozygote bearing *OfCad* deletion mutation (rr) (Table 3). When F_2_ larvae were treated with a diagnostic concentration of Cry1Ac for 7 d, 16.6% (40/241) survived. All 40 survivors of the F_2_-treated group were mutant homozygote, while the F_2_-untreated samples were divided into wild-type homozygote (ss: 25), mutant heterozygote (rs: 47) and mutant homozygote (rr: 16) (Table 3). Our results definitively revealed that the *OfCad* knockout is significantly linked with Cry1Ac resistance (Fisher’s exact test, *p* < 0.0001) in the *OfCad*-KO strain.

## 3. Discussion

Resistance to the Cry1A subfamily is reported to be associated with changes in the sequences or transcription of the cadherin gene in the midgut brush border membranes of several lepidopteran insects [17,51]. Here, we employ both in vitro and in vivo approaches to clarify whether *O. furnacalis* cadherin mediates Cry1Ac cytotoxicity and the development of resistance to Cry1Ac. Sf9 cells transfected with *OfCad* showed significant binding with Cry1Ac toxin by immunolocalization analysis and exhibited a concentration-dependent mortality effect when treated by Cry1Ac. The CRISPR/Cas9 generated cadherin knockout strain *OfCad*-KO obtained a moderate level of resistance to Cry1Ac. These results demonstrate that the *OfCad* protein is a functional receptor for Cry1Ac toxin and makes a certain contribution to Cry1Ac resistance in *O. furnacalis.*

Heterologous expression of insect cadherin protein in cell lines coupled with cytotoxicity assays is a convenient approach to investigate its response to Cry1A toxins. In this study, the Sf9 cells expressing *OfCad* protein were killed by the Cry1Ac toxin in a concentration-dependent manner, supporting that *OfCad* is a functional receptor for Cry1Ac in *O. furnacalis.* Similarly, through exogenous expression of cadherin from *Ostrinia nubilalis* (OnBt-R_1_), *H. virescens* (HevCaLP) and *H. armigera* (HaCad) in Sf9 or *Drosophila* S2 cells, their functional roles as a Cry1Ab/1Ac receptor have been well clarified [36,52,53]. However, the cadherins from two *Spodoptera* species were not involved in the Cry1Ab/1Ac toxicity. Expression of *S. frugiperda* cadherin (SfCad) or *Spodoptera litura* cadherin (SlCAD) in Hi5 cells did not induce susceptibility to Cry1Ab or Cry1Ac, respectively [43,54]. Thus, the Cry1A toxins exert toxicity in the two *Spodoptera* species and might rely on other pathway(s) independent of cadherin.

Notably, immunofluorescence detection revealed that the binding of Cry1Ac with *OfCad* protein was significant but not strong (Figure 1B), which implies that the affinity between the *OfCad* receptor and Cry1Ac toxin is limited. This directly leads to a larger toxicity concentration (654.3 nM) of Cry1Ac causing half of the cells to swell. Using the same experimental system in our laboratory, Zhang et al. [36] showed that the LC_50_ of Cry1Ac against Sf9 cells expressing *H. armigera* HaCad was 38 nM. It is reasonable to speculate that the *OfCad*-mediated Cry1Ac toxicity in *O. furnacalis* is lower than the capability of HaCad in *H. armigera*, even though the *OfCad* here showed an effective role in Cry1Ac exerted cytotoxicity. In fact, the widely studied cadherins (including the *OfCad* in the current study) are orthologous or highly homologous to the BT-R1 gene of *Manduca sexta*, which was the first identified to bind Cry1A toxins with high affinity [55,56]. Insect cadherins that bind Cry toxins belong to a subfamily of cadherin proteins; whether other member(s) of the family are involved in the Cry1A mode of action needs to be addressed in future studies.

CRISPR/Cas9 mediated gene editing technology has revolutionized research strategies for (bio)insecticide resistance and has been applied to evaluate the contribution of a candidate gene for an observed resistance phenotype. Since 2016, a series of studies elucidated the relationships between candidate cadherin genes and the Bt resistance in insects using the CRISPR/Cas9 approach. Based on available evidence, single *H. armigera* HaCad, with a 549-fold contribution to Cry1Ac resistance, is unambiguously a primary functional receptor for Cry1Ac [57] and can be identified as a major pathway for Cry1Ac against *H. armigera*. In contrast, cadherins from *Spodoptera exigua, S. frugiperda* and *T. ni* were minor or non-functional receptors for Cry1Ab/1Ac, and these knockout strains did not or only slightly reduced the susceptibility (less than 3.3-fold) of larvae for Cry1A toxin [15,43,58]. Hence, the role of cadherin in the Cry1A mode of action and their contributions to the resistance phenotype vary among lepidopteran species. The 14-fold reduction in susceptibility to Cry1Ac in the *OfCad*-KO strain, together with the results from cytotoxicity assays (a larger LC_50_ value) in this study, further revealed that *OfCad* is involved in Cry1Ac toxicity as a minor functional receptor in *O. furnacalis.*

In our previous study, the *OfABCC2* knockout strain OfC2-KO showed 8.1-fold resistance to Cry1Ac in *O. furnacalis*, compared to the background NJ-S strain [50]. In the current work, the cadherin knockout strain *OfCad*-KO obtained a similar level resistance (14-fold) to Cry1Ac toxin compared to the same original NJ-S strain. We therefore conclude that both cadherin and ABCC2 are effective functional receptors for Cry1Ac, and the relative contribution of each to Cry1Ac resistance is limited in *O. furnacalis*. Functional studies performed in insect cells or *Xenopus* oocytes indicated that co-expression of cadherin and ABCC2 from *Bombyx mori*, *H. virescens* and *H. armigera* exhibited significant synergism of Cry1A toxicity [18,19,54,59]. Disruption of the cadherin pathway alone or of the ABCC2 pathway alone slightly reduced larval susceptibility to Cry1Ac-A10s (an evolved Cry1Ac toxin) by 2.8- to 7.9-fold. However, the double mutant strain obtained more than 3700-fold resistance to Cry1Ac-A01s [15]. It is suggested that the cadherin and ABCC2 may have synergistic effects on Cry1Ac toxicity against *O. furnacalis*. It will be interesting to test whether knockout of both cadherin and ABCC2 of *O. furnacalis* will result in high-level resistance to Cry1A toxins.

Interestingly, with 82 generations of selection, a laboratory strain of *O. furnacalis* (ACB-AcR) developed 48.9-fold resistance to Cry1Ac [60]. Downregulation and mutation of a cadherin was reported to be associated with Cry1Ac resistance in the ACB-AcR strain [49]. Subsequently, by continued selection to 145 generation, a high level of resistance to Cry1Ac (3584-fold) was detected in the ACB-AcR, and gene expressions of two aminopeptidase N genes (*apn1* and *apn3*) and an *ABCG1* gene were significantly downregulated in this strain [61]. In addition, qRT-PCR indicated that there was no significant difference in transcription of the *OfABCC2* gene between the ACB-AcR and the susceptible ACB-Bts strain [61]. It would be intriguing to map Cry1Ac resistance genes in the ACB-AcR strain of *O. furnacalis* and to further determine the involvement of *OfCad* and/or *OfABCC2* in Cry1Ac resistance.

In conclusion, we confirmed that *O. furnacalis* cadherin protein is a functional receptor for Cry1Ac, and we evaluated the contribution of *OfCad* for Cry1Ac resistance phenotype. The current study also indicates that the moderate-level resistance to Cry1Ac was recessive and showed no significant cross-resistance to Cry1Ab and Cry1Fa in the *OfCad*-KO strain. Our findings provide direct evidence that loss-of-function mutation of *OfCad* confers resistance to Cry1Ac, and enables us to design molecular means for early detection, monitoring and management of Bt resistance in field populations of *O. furnacalis*.

## 4. Materials and Methods

### 4.1. Insects and Rearing

The NJ-S strain [50], used as a wild-type control, was collected from Nanjing, China, and has been reared in the laboratory over 11 years without exposure to any (bio)insecticides. The *OfCad*-KO strain is homozygous for 15.4 kb deletion of cadherin, generated from the NJ-S strain using CRISPR/Cas9 mediated genome-editing.

The *O. furnacalis* larvae were maintained on an artificial diet (primarily made from soybean and corn) at 27 ± 1 °C, 80% relative humidity (RH) and a photoperiod of 16 h: 8 h (light:dark). The pupae were collected and put into mating cages. Adults were provided with 10% sugar solution to replenish energy and allowed to oviposit on waxed papers. Egg masses were maintained in moistened plastic boxes until hatching.

### 4.2. Bt Toxins and Bioassay

Lyophilized Cry1Ac, Cry1Ab and Cry1Aa toxins (with 99.9% purity) were bought from Dr. Marianne Pusztai-Carey (Case Western Reserve University, Cleveland, OH, USA). The Cry proteins were produced using recombinant *Escherichia coli* culture and were activated with trypsin. The purity of the Cry1A proteins was determined using high-performance liquid chromatography and SDS-PAGE [62]. Cry1Fa protein, produced by recombinant B. thuringiensis acrystalliferous mutant strain HD-73^−^ harboring the cry1Fa gene, was activated and purified by the Institute of Plant Protection, Chinese Academy of Agricultural Sciences, Beijing, China [63].

Toxicological response of O. furnacalis larvae to Cry toxin was tested by a diet incorporation method. Stock suspensions of toxin were diluted with PBS (0.01 M, pH 7.4). Each Bt toxin was diluted into five to seven concentrations as test solution, and PBS was used as a control. The test solution was added to diet and mixed thoroughly until it became a smooth dough. Subsequently, the diet incorporated with toxin was dispensed into each hole of a 24-well plate (surface area per well = 2 cm^2^). When the diet solidified, one newly hatched larva was placed in a well. After 7 d of treatment in an illumination incubator with 27 ± 1 °C, 80% RH and 16 L: 8 D photoperiod, larvae were scored as dead if they weighed ≤5 mg or if they died. The data were analyzed with PoloPlus [64] to calculate the LC_50_ and its 95% confidence limits. The resistance ratio (RR) was calculated by dividing the LC_50_ value of a test strain by the LC_50_ value of the NJ-S strain.

### 4.3. Construction of OfCad Expression Plasmids

According to the whole-genome sequences of the Asian corn borer (NJ-S, our unpublished data), the full-length gDNA (*ca.* 24.1 kb) of the *O. furnacalis* cadherin (*OfCad* gene) were assembled. PCR application for ORF of *OfCad* was performed by using a pair of primers (Full_F and Full_R, Table S2). The PCR fragment corresponding to the expected size of the *OfCad* ORF sequence (about 5.2 kb) was excised from 1% agarose gels. The purified product (following the protocol of AxyPrep^TM^ DNA Gel Extraction Kit, Axygen Scientific Inc., Union City, CA, USA) was recombined with pFastBac™ HTA donor plasmid (Thermo Fisher Scientific, Waltham, MA, USA), which linearized by digestion with the *Stu* Ⅰ restriction enzyme, by using Exnase Ⅱ homologous recombinase (ClonExpress^®^II One Step Cloning Kit, Vazyme, Nanjing, China). The recombinated plasmids were sequenced by TSINGKE Biological Technology (Nanjing, China). The obtained pFastBac™ HTA-*OfCad* plasmids were extracted by AxyPrep^TM^ Plasmid Miniprep Kit (Axygen Scientific, Inc., Union City, CA, USA) and stored at −20 ℃ for future use.

### 4.4. Expression of the Recombinant Plasmids in Sf9 Cells

*Spodoptera frugiperda* Sf9 cell lines [36] were cultured in Sf-900™ III SFM medium (Thermo Fisher Scientific, Waltham, MA, USA) at 27 °C with a density of 5 × 10^6^ cells/mL and subcultured every 3 d. Log phase cells were used for infection and were seeded at a density of 2 × 106 cells mL−1, and transfected with P3 recombinant baculovirus stocks. By following the operation manual of Bac-to-Bac baculovirus expression system (Thermo Fisher Scientific, Waltham, MA, USA), purified pFastBac™ HTA-*OfCad* plasmids were transformed into E. coli DH10Bac cells to generate recombinant bacmids. Purified recombinant bacmids were used to infect Sf9 by using the FuGENE HD transfection reagent (Promega, Madison, WI, USA). P3 viral stocks were stored at 4 °C for further use. Negative control used in the current study includes cells transfected with empty bacmid (EB) and the non-transfected cells (Sf9).

### 4.5. Detection of Recombinant OfCad Expression in Sf9 Cells by Immunoblot Analysis

Immunoblot analysis was employed to detect expression of recombinant *OfCad* protein. After 2.5 d of transfection, 1 × 107 transfected cells were washed with PBS (10 mM Na2HPO4, 135 mM NaCl, 1.7 mM KH2PO4, 2 mM KCl, pH 7.4) and lysed in CytoBuster protein extraction reagent (Novagen, Madison, WI, USA). Cell lysates were centrifuged, and the membrane pellets were solubilized in buffer (2 M thiourea, 5% (*w*/*v*) CHAPS, 5 M urea, 40 mM Tris-HCl) and collected as membrane fraction. Ten micrograms of total protein was separated by 10% SDS-PAGE and transferred to PVDF filter (Millipore Corp., Burlington, MA, USA). Filters were blocked with 5% BSA in PBST (135 mM NaCl, 3 mM KCl, 25 mM Tris, 0.1% Tween 20, pH 7.4) for 2 h and then detected with anti-His TagMouse monoclonal antibodies (1:5000). After being washed, PVDF membranes were incubated with goat anti-mouse IgG labeled by HRP (Sigma-Alrich, St. Louis, MO, USA) (1:10,000) in PBST for 1 h at RT. The Thermo Scientific® SuperSignal® West Pico Chemiluminescent Substrate was used to detect blotted proteins and then photographed using a Molecular Imager Model VersaDoc 4000 (Bio-Rad, Hercules, CA, USA).

### 4.6. Immunofluorescence Localization

Immunolocalization of expressed *OfCad* proteins and Cry1Ac in baculovirus-transfected cells was evaluated by using immunofluorescence microscopy. A total of 105 Sf9 cells were transfected with recombinant viruses (MOI = 2) in 35 mm glass-bottom dishes. After 48 h, cells were washed twice with PBS (pH 7.4) and fixed at RT in 4% paraformaldehyde solution for 30 min. Fixative was washed away with three PBS (pH 7.4) washes and blocked with 3% BSA at RT for 2 h. For detection of *OfCad* expression, cells were incubated overnight at 4 °C with 1:200 diluted 6 × His Tag antibody peptide mouse polyclonal antibodies. For indication of Cry1Ac binding, cells were washed with PBS (pH 7.4) and incubated with 200 nM Cry1Ac for 1 h at RT. Cells were incubated with rabbit polyclonal anti-Cry1Ac IgG antibody (1:200 dilution) overnight at 4 °C.

Cells were washed three times with PBS and then incubated with Alexa Fluor 594-conjugated goat anti-mouse (*OfCad* protein detection) or goat anti-rabbit (Cry1Ac binding detection) antibodies (1:800 dilution) (Abcam, Shanghai, China) at RT for 1 h. Nuclei were stained by DAPI (1 mg/mL, Thermo Fisher Scientific, Waltham, MA, USA) for 30 min. Unbound conjugate was removed by three PBS washes. Cells were immediately examined under a laser scanning confocal microscope (Carl Zeiss Microscopy GmbH, Jena, Germany) with 20× objective lens, and excitation at 461 nm or 594 nm for DAPI and Alexa 594, respectively. Image acquisition and data processing of the negative controls were conducted under the same conditions.

### 4.7. Cytotoxicity Determination

Sf9 cells were seeded in six-well plates at a density of 5 × 10^5^ cell per well and transfected with recombinant virus at MOI 2 for 2.5 d at 27 °C. Cells were washed twice with fresh medium and incubated with different concentrations of Cry1Ac, diluted in fresh medium with gently shaking for 60 min at 27 °C. Cells were then washed once by fresh medium and stained by 0.4% trypan blue for 5 min. Cells were counted by a BX60 Olympus light microscope (Olympus, Tokyo, Japan). The experiment was repeated three times for each type of recombinant virus-transfected Sf9 cells.

### 4.8. Preparation of SgRNA and Microinjection

Two sgRNA target sites were visually identified in the predicted exon 4 and exon 35 of *OfCad* gene by scanning the (G)N19NGG motif (see schematic diagram showing the gene structure in Figure 3A). Two sgRNA templates were produced by two pairs of oligonucleotides (gRNA_Ex4F/4R and gRNA_Ex35F/35R, Table S2) based on a PCR assembly approach, with reference to instructions of GeneArt™ Precision gRNA Synthesis Kit (Thermo Fisher Scientific, Waltham, MA, USA). After generating sgRNA by transcription reaction, 1 uL DNase I was used to remove the DNA template, and the purified sgRNAs (sgRNA_Ex4 and sgRNA_Ex35) were stored in aliquots at −80 °C.

Fresh egg masses were collected and prepared for microinjection as following the experimental protocols of Wang et al. [50]. About 1 nL mix of sgRNA_Ex4 (300 ng/µL), sgRNA_Ex35 (300 ng/µL) and Cas9 protein (150 ng/µL, Thermo Fisher Scientific, Waltham, MA, USA) were injected into each egg with a FemtoJet and InjectMan NI 2 microinjection system (Eppendorf, Hamburg, Germany). Injected embryos were maintained at 27 ± 1 °C, 80% RH and 16L: 8D photoperiod for hatching.

### 4.9. Genotyping of the Deletion Mutant

Based on the *OfCad* gene structure and sgRNA targeting locations, a pair of special primers (Outer_F and Outer_R, Figure 1A and Table S2) was designed to detect the *OfCad* deletion mutation. If the *OfCad* is deleted, a short fragment (ca. 220-bp) of the genomic DNA is expected to be amplified by PCR with Outer_F and Outer_R primers. To identify the homozygote or heterozygote bearing *OfCad* deletion mutation, the primers (Inner_F and Inner_R, Figure 1A and Table S2) were designed to amplify an approximately 750 bp wild-type *OfCad* allele. Genotyping of the samples was determined from the banding pattern of the electrophoresis image of two different PCR products (Figure 3C), amplified by Outer_F/Outer_R and Inner_F/Inner_R primers, respectively. After the knockout strain *OfCad*-KO was established, the genotype of the individual was determined by direct sequencing chromatogram of PCR products amplified by Outer_F and Outer_R. TSINGKE Biological Technology (Nanjing, China) provided the sequencing services.

### 4.10. Inheritance Model Characterization and Genetic Association Analysis

Thirty virgin female adults of the NJ-S strain were mass crossed with 30 male adults of the knockout *OfCad*-KO strain and vice versa. Survival rates of larvae from NJ-S, *OfCad*-KO and their F1 progeny were obtained at the diagnostic concentration of Cry1Ac (1 μg/g). Applying the formula of Liu and Tabashnik [65], the dominance value (h) was calculated as: (survival of F1 − survival of NJ-S)/(survival of *OfCad*-KO − survival of NJ-S). The h varies from 0 (completely recessive) to 1 (completely dominant). For genetic association analysis, the F1 progeny were mass crossed to generate F2 (Figure 4). Newly hatched F2 larvae were treated with Cry1Ac (1 µg/g) for 7 d. The DNAs of F2-untreated individuals and F2-treated survivors were extracted for *OfCad* genotyping by the banding pattern of PCR products (Figure 3C).

## Figures and Tables

**Table 1:** Toxicity of four Cry toxins against the NJ-S and *OfCad*-KO strains of *Ostrinia furnacalis*.

Toxin	Strain	N ^a^	LC_50_ (μg/g) (95% CL)	Slope ± SE	RR ^b^
Cry1Aa	NJ-S	312	0.391 (0.320–0.455)	3.71 ± 0.52	
	*OfCad*-KO	336	1.809 (1.083–2.736)	3.21 ± 0.42	4.6
Cry1Ab	NJ-S	360	0.116 (0.074–0.177)	2.98 ± 0.36	
	*OfCad*-KO	240	0.126 (0.094–0.166)	2.07 ± 0.29	1.1
Cry1Ac	NJ-S	720	0.100 (0.069–0.136)	3.25 ± 0.43	
	*OfCad*-KO	813	1.398 (0.889–2.062)	1.93 ± 0.39	14
Cry1Fa	NJ-S	408	0.108 (0.048–0.169)	2.13 ± 0.25	
	*OfCad*-KO	312	0.185 (0.147–0.232)	2.95 ± 0.39	1.7

^a^ Numbers of larvae used in bioassay. ^b^ RR (resistance ratio) = LC_50_ *OfCad*-KO strain / LC_50_ of NJ-S strain.

**Table 2:** Inheritance of Cry1Ac resistance of *OfCad*-KO strain.

Strain	N ^a^	Survival (%)	Dominance Value (*h*) ^b^
NJ-S	96	0	
*OfCad*-KO	96	57.3	
*OfCad*-KO♀x NJ-S♂ (F_1_)	144	0	0
*OfCad*-KO♂x NJ-S♀ (F_1_′)	144	0	0

^a^ Numbers of larvae treated with 1 μg/g of Cry1Ac. ^b^ The dominance value (*h*) = (survival of F_1_–survival of NJ-S)/(survival of *OfCad*-KO—survival of NJ-S).

**Table 3:** Genetic linkage analysis of *OfCad* knockout with Cry1Ac resistance in *OfCad*-KO strain.

Strain/F_2_ Progeny ^a^	Genotype ^b^		
	*rr*	*rs*	*ss*
NJ-S			30
*OfCad*-KO	30		
F_2_-untreated larvae (*n* = 88)	16	47	25
F_2_-treated survivors (*n* = 40)	40	0	0

^a^ A total of 241 F_2_ larvae were treated with diagnostic concentration of Cry1Ac (1 μg/g); 88 F_2_-untreated larvae and 40 F_2_-treated survivors were collected for genotyping. ^b^ *ss* represents homozygote carrying wild type *OfCad* allele, *rs* means heterozygote with mutant *OfCad* allele and *rr* represents homozygote bearing mutant allele of *OfCad*.

## Supplementary Materials

The following are available online at https://www.mdpi.com/article/10.3390/toxins13090658/s1, Figure S1: Immunoblot analysis of *Ostrinia furnacalis* cadherin (*OfCad*) from Sf9 cell extracts, Table S1: Mortality of Sf9 cell lines to Cry1Ac toxin, Table S2: Primers used for PCR amplification in this study.
