# Supplementary material for: Cadherin Protein Is Involved in the Action of Bacillus thuringiensis Cry1Ac Toxin in Ostrinia furnacalis
Source: Toxins (Basel). 2021 Sep 15;13(9):658. doi: 10.3390/toxins13090658 (PMC8473148; doi:10.3390/toxins13090658)
Supplement: Supplementary file 1 [file toxins-13-00658-s001.zip › toxins-1357252-supplementary.pdf]

Supplementary Materials: Cadherin protein is involved in the action of *Bacillus thuringiensis* Cry1Ac toxin in *Ostrinia furnacalis*

Wenzhong Jin, Yuqian Zhai, Yihua Yang, Yidong Wu and Xingliang Wang

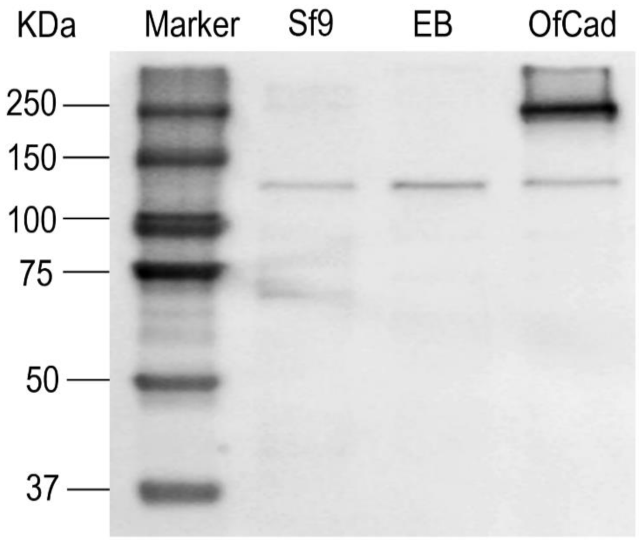

Figure S1. Immunoblot analysis of *Ostrinia furnacalis* cadherin (OfCad) from Sf9 cell extracts. Equivalent amounts (10 μg) of recombinant OfCad proteins extracted from baculovirus-transfected Sf9 cells were separated by SDS-PAGE and transferred to a PVDF membrane. Immunoblot was probed with anti-His TagMouse monoclonal antibody and HPR-conjugated goat anti-mouse. Bands indicate cross-reactivity with anti-His peptide antibody. Extracts from Sf9 (non-transfected Sf9), EB (Sf9 cells transfected with empty bacmid), OfCad (Sf9 cells transfected with full-length wild-type OfCad) are indicated above lanes. WesternC™ protein markers (Bio-Rad Laboratories, Inc. USA) were used for immunoblot experiment.

Table S1. Mortality of Sf9 cell lines to Cry1Ac toxin. Sf9 present non-transfected Sf9 cells, and Sf9 cells infected by empty bacmid or *Ostrinia furnacalis* OfCad were marked as EB and OfCad in the table, respectively.

Concentration (nM)	Cell mortality (%, mean ± SE)		
	Sf9	EB	OfCad
0	1.0 ± 0.06	2.2 ± 0.20	4.60 ± 1.20
1	1.1 ± 0.20	7.0 ± 1.64	12.1 ± 1.72
10	1.0 ± 0.10	5.5 ± 0.85	17.5 ± 1.91
100	1.2 ± 0.17	6.4 ± 0.72	22.1 ± 1.68
333	1.1 ± 0.06	4.7 ± 0.78	45.7 ± 0.36
1000	1.0 ± 0.15	6.4 ± 1.72	61.9 ± 2.34
2000	1.0 ± 0.20	6.4 ± 0.85	77.8 ± 2.66
4000	1.2 ± 0.15	5.9 ± 0.20	86.9 ± 1.46

Table S2. Primers used for PCR amplification in this study. The underlined sequence represents infusion homology arms.

Primer Name	Primer Sequences (5′ to 3′)
Full_F	GGATCCGGAATTCAAAGGCCTTATGGGGGTTGAGAGGTTCTTC
Full_R	TGAGCTCGTCGACGTAGGCCTTTACTTTGGGTGCGGCTGTG
sgRNA_Ex4F	TAATACGACTCACTATAGGCCAGCCCAGAGCCCAGCT
sgRNA_Ex4R	TTCTAGCTCTAAAACAGCTGGGCTCTGGGCTGGC
sgRNA_Ex35F	TAATACGACTCACTATAGCCACGCATGGCAACAACTT
sgRNA_Ex35R	TTCTAGCTCTAAAACAAGTTGTTGCCATGCGTGG
Outer_F	GTTGGCATCGAATCATCACCC
Outer_R	GCCGAATGACGGAGTCGGGTT
Inner_F	CACGATCACCGACCGAGACAT
Inner _R	AGTGAATGACGGAGTCGGGTT
